# In Vivo Analysis of Extract of Leaves of Mistletoe as a *Benalu Duku*: Clinical Chemical Value Associated with Histopathology, Liver, Kidneys, and Lethal Dose Determinate

**DOI:** 10.1155/2022/1182866

**Published:** 2022-12-12

**Authors:** Mochamad Lazuardi, Chi-Hsien Chien, He Jie-Long, Pramyrtha Hestianah Eka

**Affiliations:** ^1^Faculty of Veterinary Medicine, Universitas Airlangga, Mulyorejo Road “C” Campus, Surabaya 60115, Indonesia; ^2^Department of Post-Baccalaureate Veterinary Medicine, Asia University, Taichung City 41354, Taiwan

## Abstract

The active compounds from the leaves of *Dendrophthoe pentandra* L. Miq., also known as, *Benalu Duku* (in Indonesia), are known to contain progesterone-like compounds (PLCs). This study aims to determine the effect of giving a single dose of PLCs on liver and kidney function in rats and the dose limit that causes the death of experimental animals. The PLCs were analyzed for chemical and physical characterization and compared to a pure standard of progesterone using HPLC, IR spectrometry, thermogravimetry, and NMR. The research was carried out in two sections. In section one, thirty-five healthy adult male rats were divided into six experimental groups and a control group of five rats each. The groups received, respectively, 50 to 75 mg/kg of PLCs (i.p.). The control group was given a 0.5 mL Aqua Pro injection. Alanine aminotransferase, aspartate aminotransferase, creatinine, and blood urea nitrogen were assessed using the clinical chemistry of blood serum analysis. Cell disruptions were analyzed to determine the degeneration effects of PLCs on the liver and kidney in the experimental and control groups. In section two, thirty healthy adult male rats were divided into 6 groups, each group of 5 rats, and injected with PLCs at a dose of 0.9–2.1 g/kg BW, followed by a lethal dose test. The control groups were available for 5 individual rats at 0 g/kg BW of PLCs. Our findings indicated that PLCs have a similarity chemical and physical characterized each other compounds, then the following administration of 50 to 75 mg/kg of PLCs did not affect the parameters of clinical chemistry. Histopathology analysis of the liver and kidney revealed normal subcellular levels in the experimental group, with the nonlethal dose at 0.9 g/kg BW.

## 1. Introduction

The principle of Sustained Development Goals from The World Health Organization in point 3 of health at the subtopic of medicine (traditional and complementary herb medicine) revealed the useful herb medicine administration in human cases. *Dendrophthoe pentandra*, also known as mistletoe, or *Benalu Duku* (BD) in Indonesia, is a parasitic herb that grows on the host plant *Lancium domesticum* Corr. Mistletoe leaves exert antiproliferative effects on cancer cells in vitro and in vivo, as well as myeloma cells [1]. BD leaves have the potential as an anti-coronavirusdisease-2019 regimen with mechanisms such as cancer cell antiproliferation and antiviral against influenza viruses in cats and dogs by damaging the protein envelops of the virus and making a stimulated antibody system [2]. The host plant is well distributed in many countries worldwide and is known to grow well in tropical and subtropical countries [3, 4]. Crude methanol extract of BD leaves contained 7.622% (w/w) essential amino acids from 4.384 mg of pulverized leaves before extraction [5]. Several reports between 2009 and 2010 showed that BD plants contained hydroxyl, carbonyl, and amine functional groups, double-bond aliphatic chains, various alkaloids, flavonoids, polyphenols, terpenoids, and steroids [6]. The ethanol and methanol extracts of BD leaves were known to contain androgenic hormones of progesterone-like compounds (PLCs) at approximately 66%. The remaining 34% androgenic compounds were known as follows: medroxyprogesterone acetate, megestrol acetate, and dydrogesterone [7]. However, the active substances from the crude extract of BD leaves have not been proven safe for animals, especially for liver as a metabolism organ and kidneys as a filtration organ. Therefore, this study aimed to investigate the degeneration effects of crude methanol extract of BD leaves on the liver and kidney by investigating the clinical chemistry parameters such as alanine aminotransferase (ALT), aspartate aminotransferase (AST), creatinine, and blood urea nitrogen (BUN). So, it is also necessary to know the 50% dose limit that causes death. The results of additional studies also analyzed the possibility of finding a degenerative effect in rats due to PLCs administration and the dose limit that did not cause death.

This research stage will use a new modification by elaborating three parameters, namely, clinical chemistry studies, analysis of liver and kidney damage, and analysis of possible risk of death due to the impact of toxicity of polar solvent mistletoe extract. The new modification will be able to describe the impact of toxicity from the subclinical to the clinical level. Additionally, PLCs were isolated from the crude extract using high-performance liquid chromatography (HPLC) and then profiled to obtain the specific physical and chemical characteristics of bioactive substances.

## 2. Materials and Methods

### 2.1. Plants Collection and Progesterone Standard

The plant material (leaves) was pressed and transferred for identification and authentically to the herbarium of Botanic Research Center, Cibinong Science Center, Raya Jakarta-Bogor, Km.46 Cibinong 16911, Bogor-Indonesia, under letter at date 02-03 2019 as a *Dendrophthoe pentandra* L. Miq., respectively. BD leaves growing well on the 3-year-old *L. domesticum* Correa were obtained from Muaraenim, Indonesia (geographical coordinates at 3° 39′ 0″ South, 103° 48′ 0″ East). The leaves were collected at the beginning of the rainy season from December 2018 to December 2019. The certified reference material (CRM) of progesterone was obtained from Fluka Corporation, catalog number SZBA321XV.

### 2.2. Instrument Analysis

Prestige 24i Automated Analyzer from Cosmos Biomedical Ltd. (2 Coronation Lane, DE12 7XB, Swadlincote, United Kingdom) was used to analyze the serum levels for AST, ALT, creatinine, and BUN. HPLC semipreparation UV-photodiode array (PDA) detection was performed using Shimadzu LC-6AD pump with a DGU-20A5 degasser, communication module-20 A, and PDA detector SPD-M20A with an FRC-10A fraction collector. The UV detector for HPLC was set at 254 nm with a reverse-phase ODS C_18_ column for analysis or semipreparation column Zorbax Eclipse XDB-C_18_ at length of 9.4 × 250 mm, 5 *μ*m. The mobile phase was methanol: water at a ratio of 70 : 30 [5].

Thermogravimetric analysis was performed using a Mettler analyzer. The temperature was maintained between 25°C and 150°C, and the minimum weight was set as 400 mg. The operating conditions were adjusted at a 30 min interval, and the temperature was gradually increased at a rate of 10°C/s.

The infrared (IR) analysis was performed using the diffuse-reflectance spectroscopy method or diffuse-reflectanceFourier-transform infrared (FT–IR) spectroscopy (Shimadzu IR Tracer-100;https://www.shimadzu.com/an/molecular_spectro/ftir/irtracer/irtracer2.html).

Proton nuclear magnetic resonance (^1^H NMR) method was performed using the JEOL Resonance Shimadzu ECS-400 instrument after diluting the samples with water D_4_ grade methanol. The parameters were processed at a field strength of 9.389766 and a frequency of 400 MHz. The duration of each analysis was 1.6384 s. The results analysis of ^1^H NMR were compared to CRM or referred to https://go.drugbank.com/spectra/nmr_one_d/1582.

### 2.3. Plant Extraction and Animal Experimentation

Approximately 2 kg of pulverized BD leaves were percolated with methanol at a proanalysis grade. Then, approximately 20 g of crude extract was vacuum-dried and evaporated with nitrogen gas at 40°C. The crude extract was prepared to obtain steroid compounds and then the following purity by semipreparation of HPLC for obtaining PLCs [5]. The PLCs were identified and characterized using thermogravimetric analysis, FT-IR spectroscopy, and NMR by comparing them to the CRM. Following step, determined PLCs as a steroid compound using the Liebermann–Buchard test by dissolving PLCs in chloroform (w/v) and adding 1–3 drops of a solution of acetic anhydride and concentrated sulfuric acid to produce a colour change from blue to red [8]. The PLCs were processed in sterile dosage form as injection products for all the experimental animals [9].

The rats (certified by a veterinarian to be healthy) were obtained from a local breeder in Surabaya, Indonesia. Animal ethics clearance was obtained from the Faculty of Veterinary Medicine, Universitas Airlangga (approval number: KE: 1.KE.132.07.2018) on July 31, 2018.

The sample size was computed using the (1) below with the following: assumed {*Z*_1_ − (*α*/2)} at 1.96, a significance of 0.05; *Z*_*β*_ at 1.645 by error limit of 5%; *d* at 3.62; S*a* at 1.7, and S*b* at 1.4 [5]. The *N* value was rounded to 5 rats for each treatment dose; thus, the control group should consists of five sets of data.

(1) $$\begin{array}{c} N=\frac{{\left[\left(Z_{1}-\alpha /2\right)+Z_{\beta }\right]}^{2}}{\left(d_{2}\right)/{\left(S_{a}\right)}^{2}+{\left(S_{b}\right)}^{2}}\text{.} \end{array}$$

Total animals experimental used sixty-five rats at two stages research design. The rats used at two sections research stage, as follows: section one (*n* = 35) for analysis clinical chemistry included analysis degenerative effect in liver kidney, and section two (*n* = 30) for analysis determined lethal dose of PLCs.

### 2.4. Clinical Chemistry Examination

The rats were divided into an experimental group (*n* = 30) and a control group (*n* = 5), and then divided into six groups of five rats each. The rats in the experimental groups were injected intraperitoneally (i.p.). Administered a single dose of methanol extract from BD leaves as a research analyte. Group 1 received 50 mg/kg BW of analytes, groups 2 received 55 mg/kg BW of analytes, group 3 received 60 mg/kg BW of analytes, group 4 received 65 mg/kg BW of analytes, group 5 received 70 mg/kg BW of analytes, and group 6 received 75 mg/kg BW of analytes. One group as a control group of 5 individual rats was given 0.5 ml i.p. Aqua Pro injections [10, 11]. Determination of the dose of 50–75 mg/kg BW is based on the usual standard dose that has been patented No. IDP000063522. Granted, on the date of October 15, 2019 (https://pdki-indonesia.dgip.go.id/search?type=patent&keyword=Benalu+duku&page=1).

Subsequently, general anesthesia was administered, namely ketamine at 87 mg/kg BW (subcutan). From the tail vein of rats, approximately 1 mL of blood was collected for ALT, AST, creatinine, and BUN analyses. ALT, AST, creatinine, and BUN levels in the control group were measured in the collected blood serum from the rats after administration of i.p. sterile, blank substances. The serum levels of AST, ALT, creatinine, and BUN were detected by the Prestige 24i Automated Analyzer following the manufacturer's standard clinical chemistry examination procedures.

At the beginning of the clinical chemistry examination, partial validation was carried out, namely, sensitivity (quantification limits), linearity, repeatability, and reproducibility (referring to [12]), then the ALT, AST, creatinine, and BUN examinations were carried out below. The 100 *μ*L serum samples were added to 1 mL of kit reagent for ALT and AST (Sigma-Aldrich, Corp., US, catalog numbers MAK052-1KT and MAK055-1KT), shaken well for 5 minutes, and incubated for 2 minutes at 37°C. In the next step, ALT and AST samples were added with 200 *μ*L indicator reagent and incubated for 5 minutes at 37°C). The rate of decrease in the concentration of ALT and AST in the samples was measured photometrically at 340 nm. The 600 *μ*L of creatinine kit (Sigma-Aldrich, Corp., US, catalog number MAK080-1KT) was added to 14 *μ*L of serum samples, shaken well, and incubated for 10 min at 20–25°C. Then, add the indicator reagent in 200 *μ*L. Creatinine reacted with alkaline picrate as an indicator reagent, forming a red complex that was measured photometrically at 492 nm. The 400 *μ*L of Kit BUN reagent (Sigma-Aldrich, Corp., US, catalog number MAK006-1KT) was added to 100 *μ*L of indicator reagent and shaken well for 5 minutes. In the next step, all reagents were added to 5 *μ*L of serum sample and observed. BUN in the sample reacted with o-phthalaldehyde in an acid medium, forming a coloured complex that was measured photometrically at 510 nm.

### 2.5. Histopathology Procedures

The rats in the experimental groups of section one will be sacrificed using the cervical decapitation method under light anesthesia with isoflurane (5%) as an inhalant anesthetic (pharmaceutical grade) using modified nebulizer equipment. Thick sections (3–5 mm) of the liver and kidney were fixed overnight in neutral-buffered formalin 10% fixative, then gradually dehydrated by alcohol as follows: 70%, 80%, 90%, 96%, and absolute alcohol. In the next step, thick sections of organs were cleansed with xylol solution 3 times and embedded in paraffin (63°C). The embedded organs were blocked by paraffin and placed on the cassette holders. The blocked organs were prepared by microtome with a Sakura Finetek Accu-cut SRMM (Japan Co., Ltd.). Results of cutting are placed on the object glass and dried in hot plates for 30 minutes. The slides were removed from the paraffin content by washing with xylol 3 times. The slides were cleaned by the gradual method every 4 minutes using alcohol with decreasing concentrations, as follows: absolute alcohol: 90%, 80%, and 70%. The slides were rinsed with aqua distillate, then stained with the haematoxylin and eosin (H&E) method using Mayer's Hemalum Solution (Merck Corp., Germany, catalog number HX68597049). The last step was to cleanse with aqua distillate and then add with Eosin stain. The histopathology slides were ready for examination after adding the cover glass [13]. Examination of the slides was performed using a Nikon Eclipse E100 microscope at magnifications of 400x and 1000x. Criteria observation as follows: In one field of view at 1000x magnification and moving like the letter S, there were signs of cell death, such as no nucleus or a nucleus in an eccentric region; granules in the cytoplasm were not visible; cells were not visible; and dye had entered the internal cells.

### 2.6. Determine Lethal Dose

The rats in section two were thirty individual healthy adult male rats divided into 5 experimental groups for a serial lethal dose test as follows: 0.9, 1.2, 1.5, 1.8, and 2.1 g/kg BW twice a day (i.p). In addition, one group consisted of five experimental animals as a control group and injected i.p aqua pro injection blank compounds twice a day. The reason for determining the serial dose as described above was based on the fact that the toxic content test of natural products ranges from 6-7 times the maximal dose [14]. Observations were carried out for 5 days after the injection.

### 2.7. Statistical Analysis

The results of statistical tests were assessed using the Statistical Package for Social Sciences (SPSS) for Windows version 24.0, at 5% significance. ALT, AST, creatinine, and BUN levels were compared between the experimental and control groups using the analysis of variance. Determination of the lethal dose was achieved using probit analysis of SPSS 24.0 by significance level for the use of heterogeneity at 0.05.

## 3. Results

The peaks of PLCs were observed at retention times of 6.500 to 7.000 min in the HPLC chromatogram, and the peak of CRM was observed at 6.642 min. The separation of isolates using analytic columns showed very few active compounds because the capacity of the analytical column is very small (approximately 20 *μ*L). However, the analytical column has the following advantage: the exact time when PLCs are separated is known because it separates specific compounds. The semipreparative HPLC can be used for separating compounds, but PLCs cannot always be perfectly separated since PLCs are separated by a relatively separate column on a stable retention time condition [15].

**Figure 1:**
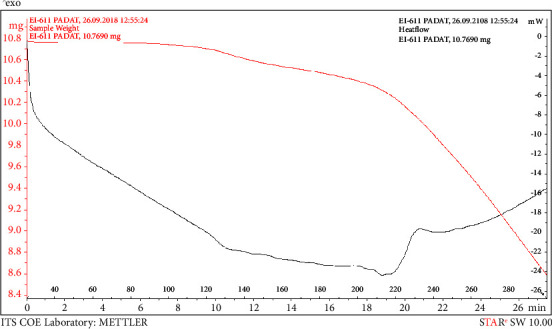
Thermogravimetric analysis of the progesterone-like effect starting with a weight of 10.769 mg (red line) with the effect of hot temperature; each heat flow of 20°C (black line), and the sample degradation starts at 110°C.

**Figure 2:**
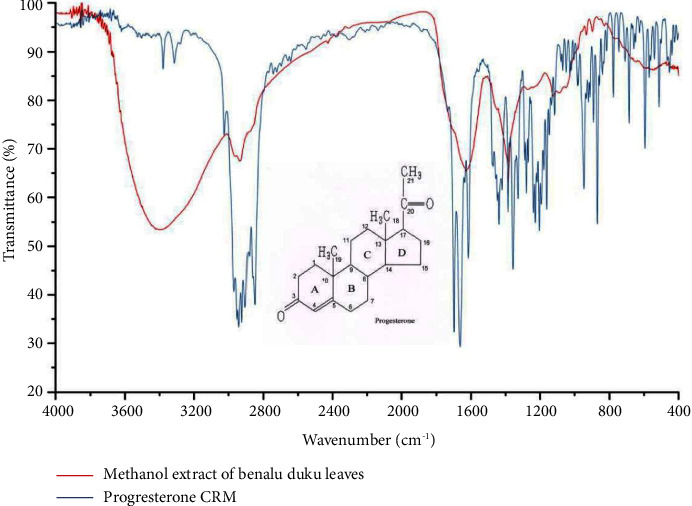
The infrared spectrum of isolated methanolic crude extract of *Benalu Duku* leaves (blue line) and CRM of progesterone (red line) at wave number 4000 to 400 cm^−1^ via the diffuse-reflectance infrared Fourier-transform method using the Shimadzu IR tracer-100.4

**Figure 3:**
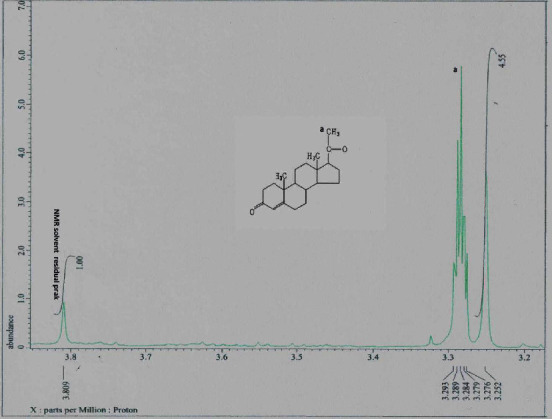
The analysis of proton NMR of carbon atom number 21 of PLCs (A) from separation extract *Benalu Duku* leaf performed using the JEOL resonance Shimadzu ECS-400 instrument after the samples were diluted with D_4_ grade methanol. Parameters were processed at a field strength transmittance of 9.389766 with a 400 MHz frequency. The duration of each analysis was 1.6384 s.

**Figure 4:**
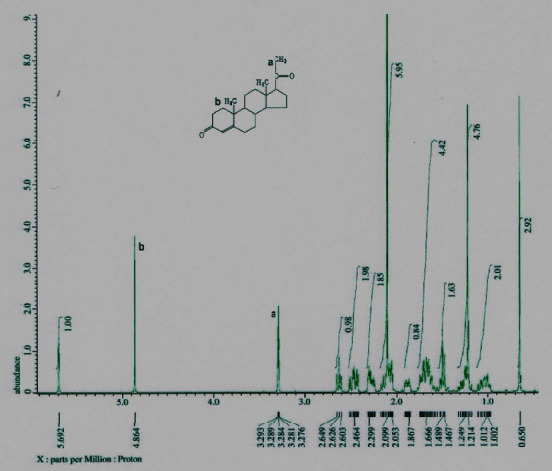
Proton analysis CRM of progesterone dissolved in methanol-D_4_ with carbon atom numbers 21 (A) and 19 (B) using the JEOL Resonance Shimadzu ECS-400 instrument after the samples were diluted with D_4_ grade methanol. Parameters were processed at a field strength transmittance of 9.389766 with a 400 MHz frequency. The duration of each analysis was 1.6384 s.

The melting point of PLCs (140°C) isolated from crude methanol extract was close to that of the CRM (120°C). Differences in melting points occurred because of slight differences in the core of PLCs, which may be bound to other elements of the sample matrix.  Figure 1 shows the melting point at 140°C. Thermogravimetric analysis indicates that at 105°C an early decomposition was stated. The decomposition of PLCs decreased the strength of the pharmacology effect by half of its initial strength. FT-IR spectroscopy results showed that PLCs had functional groups identical to CRM functional groups.  Figure 2 shows that the spectrum at wave number 850 is the same functional group. Proton analysis of carbon ring number 21 in PLCs identified the subscript of a as a proton of CRM (Figure 3). The proton carbon numbers 21 and 19 of CRM progesterone are presented in Figure 4.

The validation method for serum analysis of AST and ALT was presented as follows: the limit of quantification was obtained at 1.5 U/L and 2.3 UL, and linearity (*R*^2^ > 0.98) was obtained up to 500 U/L and 200 U/L. The repeatability of ALT and AST using the run-to-run method (*n* = 20) in low and high concentrations were (means ± SD) 23.98 ± 0.88 U/L (CV 3.70%), 170.72 ± 0.23 U/L (CV 3.93%), 48.12 ± 1.56 U/L (CV 3.24%), and 115.03 ± 0.75 U/L (CV 0.65%). The reproducibility of ALT and AST using the day-to-day method (*n* = 20) in low and high concentrations were obtained (mean ± SD) as follows: 143.95 ± 2.25 U/L (CV 1.56%), 230.20 ± 1.72 U/L (CV 0.75%), 45.25 ± 1.63 U/L (CV 3.6%), and 118.12 ± 0.78 U/L (CV 0.66%). The validation method for the analysis of creatinine and BUN was presented as follows: limit of quantification at 0.001 mg/dL and 0.002 mg/dL, linearity (*R*^2^ > 0.99) at up to 2.05 mg/dL and 35.05 mg/dL. The repeatability of creatinine and BUN were determined using the run-to-run method (*n* = 20) at low and high concentrations (mean ± SD) 0.50 ± 0.02 mg/dL (CV 4.00%), 150 ± 3.08 mg/dL (CV 2.05%). The reproducibility of creatinine and BUN at low and high concentrations using the day-to-day method (*n* = 20) was (mean ± SD) 0.45 ± 0.01 mg/dL (CV 2.22%), 160.02 ± 2.08 mg/dL (CV 1.30%), 16.03 ± 0.58 mg/dL (CV 3.62%), and 35.05 ± 0.89 (CV 2.54%).

**Figure 5:**
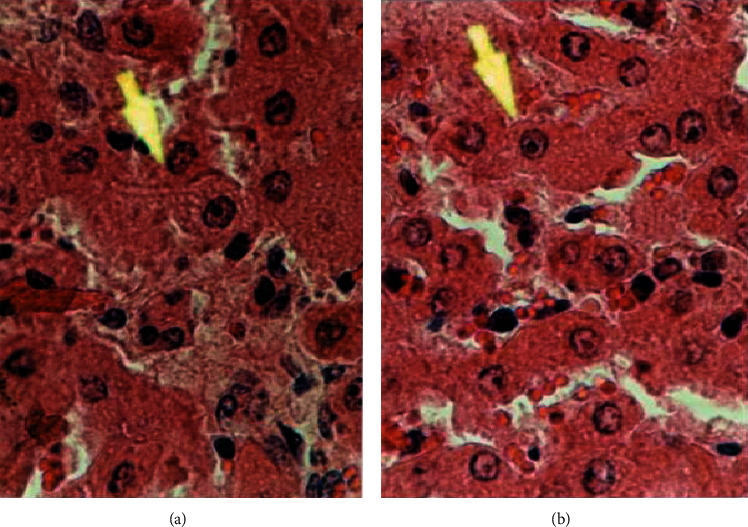
The structure of liver cells in the experimental group after administration of progesterone-like compounds at 75 mg/kg body weight (a) was the same as in the control group (b). H&E staining, 1000×.

**Figure 6:**
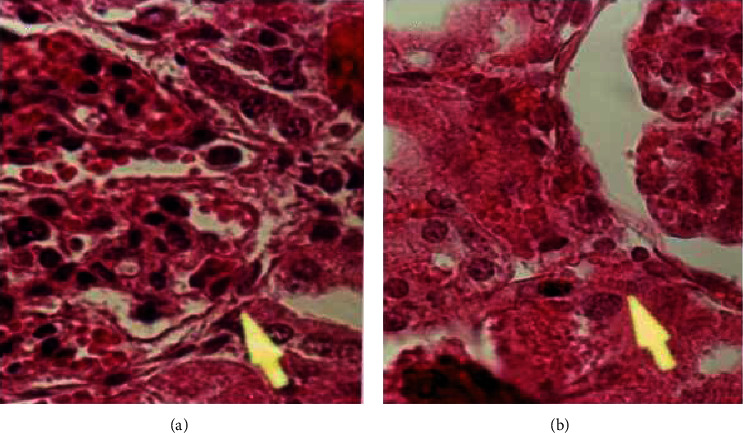
The structure of kidney cells in the experimental group after administration of progesterone-like compounds at 75 mg/kg body weight (a) was the same as in the control group (b). H&E staining, 1000×.

The result research of AST, ALT, creatinine, and BUN in experimental groups were 113.000–133.000 U/L at CV (%), 1.618–30.270; 60.000–79.800 U/L at CV (%), 2.750–13.177; 0.670–0.974 mg/dL at CV (%), 1.492–25.770; and 18.400–28.880 mg/dL at CV (%), 2.190–12.872, respectively (Table 1). Histopathology evaluation of the liver and kidney in the experimental groups was presented in Figures 5 and 6. The overview of structure cells of the kidney and liver of the experimental groups, after giving 75 mg/kg BW of PLCs, were identical to the control groups.

The results of the research for the analysis of the 50% end point to obtain the lethal dose are presented in Table 2. Starting with a dose of 1.2 g/kg BW, 20% of rats died, followed by doses of 1.5 and 1.8 g/kg BW, respectively, for 40%, and a dose of 2.1 g/kg BW for 60%. It was found that 50% of rat deaths occurred at a dose of 1.853 g/kg BW.

## 4. Discussion

Extraction of active compounds, namely PLCs, from BD leaves, was successful using the new method of HPLC in isocratic models [16]. The retention times of PLCs showed that the analyte was presented at 6.892 min and the CRM of progesterone at 6.642 min. The shift of the chromatogram peak, approximately 0.25 min between PLC peaks and CRM peaks, was tolerable. Thermogravimetric analysis showed that the decrease in weight of PLCs started at 80°C. Subsequently, the compounds decomposed at an increased temperature, and physical damage was initiated at 110°C. Progesterone degradation occurs at temperatures of >100°C (Figure 1). Decomposition occurs at a temperature of >110°C with impure PLCs [17]. The purity of the active compounds determined by FT-IR, especially in the range of 1400 to 1600 cm^−1^, was similar to that of heterocyclic progesterone at rings AB and CD. The characteristics of PLCs observed by FT-IR were as follows: 1122.57 cm^−1^ (80.815%T) as C_5_H_7_ ring D bound with carbon atoms 20 and 21; 1384.89 cm^−1^ (63.101%T) as rings C and D bound to carbon atom 18; 1629.85 cm^−1^ (65.360%T) as rings B and C; 2933.73 cm^−1^ (67.437%T) rings A and B; and 3396.64 cm^−1^ (53.436%T) dominant vibrations as hydroxyl compounds bonded to ring of A. The specific IR absorbance of PLCs was similar to CRM (indicated by the blue line). The difference in the percentage of transmittance might be correlated with the purity level of isolates, as presented in Figure 2. The NMR spectroscopy results revealed a similarity between the carbon atom proton number 21 (a) at 3.289 ppm in PLCs and the carbon atom proton number 21 in CRM (Figure 3). The carbon atom proton number 19 (b) at 4.864 ppm in CRM and compared to spectra proton NMR library in the drug Bank were absent in PLCs (Figure 4). These findings show that the purity of PLCs, as a composite product, is not equivalent to that of CRM.

The results of the validation method on clinical chemistry examinations show that the quantification limit was quite low and very sensitive, even though the reading technique uses photometrics. The linearity of the detector response to the sample concentration has a strong relationship with the measurement range. With the validation of the method on repeatability and reproducibility, the average CV was less than 5%. These results indicate that the reading device and the sample preparation process up to the time of reading on the photometric device have good repeatability, and they conform to the system suitable test (SST) principle [18].

The AST and ALT values in the experimental groups treated with PLCs from the BD leaf extracts, at doses ranging from 50 to 75 mg/kg BW, did not increase compared with those in the control group (Table 3). This finding indicated that PLCs can be safely administered at up to 75 mg/kg BW with no toxic liver effects. However, the kidney organs were prudent at a starting dose of >65 mg/kg BW (*P* < 0.05). Creatinine and BUN values in the experimental groups were significantly elevated compared with the values in the control groups but still within normal ranges for rats (normal serum creatinine and BUN of rats were 0.250–3.050 mg/dL and 15.000–28.300 mg/dL, respectively) [19–21]. Thus, the isolated PLCs were predicted to contain other compounds that would disrupt the kidney cells, namely, triterpenoids and triterpenoids saponins [22]. The dominant compounds were solvents that were used in the first step of BD leaf extraction, namely, methanol. Methanol extraction performed at concentrations of 2%-3% may lead to necrosis in the eukaryotic cells. However, the current research method reports that the solvent evaporates due to the heating process in a water bath at 40°C. Histopathology of the liver and kidneys did not show subcellular degeneration after PLC administration in the experimental group, indicating that the liver and kidneys of the experimental group had the same structure as the control group. The experimental and control group cell showed a noneccentric nucleus and cytoplasm containing the Golgi apparatus, even after administering 75 mg/kg BW of PLCs (Figures 5(a) and 5(b)). The common toxic effects on cells include an eccentric nucleus and the absence of granules; apparatuses include the Golgi apparatus. Additionally, red blood cells would be accumulated in the central hepatic vein, which was not found in the liver samples from any experimental groups (Figure 5(a)). The disruption effects on kidney cells were as follows: an eccentric nucleus and no granules in the cytoplasm. Necrotic cells were stained with H&E staining. Bowman's capsule showed an accumulation of red blood cells [23–26]. As shown in Figures 6(a) and 6(b), cells in the category of degenerative cells were not found in the kidney cell organs of the experimental or control groups. The degeneration effects of the methanol extract from BD leaves on the liver and kidney tissue that were observed at 75 mg/kg BW were not severe (borderline to mild); therefore, this extract can be used in other species, such as poultry, sheep, cows, and cattle. In this study, we did include the other mentioned species (poultry, sheep, cows, and cattle), but viewing the rats will be applied to other species. We were unable to analyze the toxic concentration of PLCs in the rat plasma or other organs than livers and kidneys, which was our study limitation. In Table 2, it is known that the safe dose of administration until no death occurs is 0.9 g/kg BW. At this dose (below the 50% end point for a lethal dose of 1.853 g/kg BW), it is probably the maximum body limit for mice to receive PLCs. However, if you want to convert it to other animal species, you can use the body surface area theory or the allometric scaling theory. The pharmacokinetic theory explains the administration of the maximum dose extravascularly (such as i.p.), which has not resulted in the phenomenon of death in clinical subjects because it will begin with the phenomenon of toxicity, even though the risk of toxicity is smaller than the risk of an intravascular injection. This is due to the phenomenon of the first pass effect, which is often found in the extravascular administration of drugs.

From a theoretical point of view, it can be predicted that the larger the animal species, the more resistant it is to organic solvents, such as methanol, which causes cell degeneration. However, the opposite is true, i.e., the more animal species that lead to small species of laboratory animals, the lower the resistance to the methanol solvent. Thus, laboratory animals, such as rabbits and guinea pigs, will not tolerate it and cause an increase in the body's clinical chemistry parameters. An initial screening method, namely, a toxicity test on tissue cultures of eukaryotic cell lines, can be used to test the sensitivity of eukaryotic cells to compounds classified as disruptive. This method is widely used in the drug industry to reduce the use of experimental animals.

Poultry species, such as seed-eating birds and chickens, are more sensitive to compounds that cause liver and kidney cell disruption, such as methanol. However, poultry species, such as meat-eating birds, are relatively more resistant to the effects of methanol since they have a neutralizing system for disruptive compounds in their bodies. However, the body's ability to neutralize disruptive compounds is limited. Thus, the body will not be able to neutralize the disruptive compounds that have already entered if the concentration of disruptive compounds is high.

All compounds that disrupt the liver and kidney cells, such as polar organic solvent compounds such as methanol, ethanol, propanol, and butanol, in limited quantities can be neutralized by the body [27]. Therefore, if wild animals or exotic carnivorous species accidentally eat something that contains a limited level of disruptive compounds, the body can neutralize it. Disruptive compounds are found, especially in the remains of grains that undergo fermentation to produce R-OH elements in the composition of the fermented compounds.

Disruptive compounds will also always be present if the environment of animals is polluted by industrial chemical components. This often happens in countries with high levels of hazardous chemical contaminants of industrial origin as well as contamination from hazardous chemical waste from households. Hazardous chemical contamination compounds can also come from semipolar solvents, such as acetone, ethyl acetate, and chloroform, or nonpolar solvents, such as diethyl ether [28]. These contaminants can be easily found, especially in laboratory or hospital waste disposal sites. These disruptive compounds, in limited quantities, can be neutralized in nature but will be toxic to the environment in high amounts. Thus, the One Health concept is indispensable in the management of nature for all animal species that live in the wild to be free from the influence of disruptive compounds.

This research was carried out by combining the methods of clinical chemistry analysis, liver and kidney damage, and the risk of death of experimental rats, so it can be concluded that we have succeeded in assessing the hazardous components of the solvent extract of *Dendrophthoe pentandra* (L.) Miq. leaf extract.

## 5. Conclusion

In conclusion, isolates of PLCs obtained from methanol extraction of BD leaves that grow well in *L. domesticum* at a dose of 50–75 mg/kg BW were safe for treatment use. PLC administration at >75 mg/kg BW carries a risk of necrosis of the kidney. Additionally, the dose of PLCs causing early death was found to be at 1.2 g/kg BW, thus the nonlethal dose was found to be up to 0.9 g/kg BW.

## Figures and Tables

**Table 1:** The parameters of the clinical chemistry of rats after giving progesterone-like compounds single-dose administrations in trial groups compared to control groups after giving 0.5 mL of Aqua Pro injection.

Groups	Clinical chemistry assessment	No. of rats					Coefficient variation (%)
		1	2	3	4	5	
Control group	AST (U/L)	170	195	90	95	100	37.586
	ALT (U/L)	80	110	70	50	60	31.111
	Creatinine (mg/dl)	0.81	0.88	0.65	0.64	0.72	14.054
	BUN (mg/dL)	18.50	18.90	19.23	19.26	19	1.618

Group I (50 mg/kg BW)	AST (U/L)	135	120	120	180	110	1.618
	ALT (U/L)	80	75	80	80	79	2.751
	Creatinine (mg/dL)	0.67	0.66	0.68	0.66	0.68	1.492
	BUN (mg/dL)	17.50	19	17.50	19.50	18.50	4.859

Group II (55 mg/kg BW)	AST (U/L)	100	125	120	100	125	11.352
	ALT (U/L)	75	75	80	70	70	5.653
	Creatinine (mg/dL)	1.41	0.78	0.88	0.85	0.95	25.770
	BUN (mg/dL)	24.60	28.90	27.30	28.50	27.40	9.310

Group III (60 mg/kg BW)	AST (U/L)	125	140	120	125	125	5.971
	ALT (U/L)	75	60	80	70	80	11.462
	Creatinine (mg/dL)	0.75	0.77	0.68	1.02	1.01	4.532
	BUN (mg/dL)	25.20	24.40	26.60	31.50	32.10	12.872

Group IV (65 mg/kg BW)	AST (U/L)	145	120	111	114	112	11.784
	ALT (U/L)	70	80	81	84	84	7.220
	Creatinine (mg/dL)	0.76	0.69	0.71	0.68	0.69	4.533
	BUN (mg/dL)	17.80	18.40	19.70	19.30	19.60	4.356

Group V (70 mg/kg BW)	AST (U/L)	110	170	80	95	110	30.270
	ALT (U/L)	50	55	60	65	70	13.177
	Creatinine (mg/dL)	0.77	0.89	0.85	0.71	0.79	8.728
	BUN (mg/dL)	25.70	26.70	25.40	26.50	26.50	12.190

Group VI (75 mg/kg BW)	AST (U/L)	120	115	115	110	123	4.336
	ALT (U/L)	70	81	75	75	83	6.790
	Creatinine (mg/dL)	0.69	0.67	0.76	0.82	0.94	14.05
	BUN (mg/dL)	27.60	28.80	28.70	29.50	29.80	2.954

**Table 2:** Analysis of lethal dose of progesterone-like compounds (PLCs) as bioactive isolates from the leaves of *Dendrophthoe pentandra* L. Miq. in healthy adult male *Rattus norvegicus* at serial doses (twice a day, intraperitoneal).

Dose (g/kg body weight)	Rats (lethal/nonlethal)	Dose 50% end point (g/kg body weight)
0	0/5	1.853
0.9	0/5	
1.2	1/4	
1.5	2/3	
1.8	2/3	
2.1	3/2	

**Table 3:** Analysis of various parameters of clinical chemistry between groups.

Parameters of clinical chemistry of rats	M ± SD							*P*
	Control groups	Group 1	Group 2	Group 3	Group 4	Group 5	Group 6	
AST (U/L)	130 ± 48.862^a^	133 ± 27.749^b^	114 ± 12.942^c^	127 ± 7.583^d^	120.40 ± 14.188^e^	113 ± 34.205^f^	116 ± 5.030^g^	0.823
ALT (U/L)	74 ± 23.022^a^	78.80 ± 2.168^b^	74 ± 4.183^c^	73 ± 8.367^d^	79.8 ± 5.762^e^	60 ± 7.906^f^	76.80 ± 5.215^g^	0.093
Creatinine (mg/dL)	0.74 ± 0.104^a^	0.67 ± 0.010^b^	0.974 ± 0.251^c^	0.706 ± 0.032^d^	0.706 ± 0.032^e^	0.802 ± 0.070^f^	0.776 ± 0.109^g^	0.020
BUN (mg/dL)	18.978 ± 0.307^a^	18.40 ± 0.894^b^	26.541 ± 2.471^c^	27.96 ± 3.599^d^	18.96 ± 0.826^e^	26.16 ± 0.573^f^	28.88 ± 0.853^g^	0.000

^a,b,c,d,e,f,g^Values within a row of AST and ALT between groups were not different (*P* > 0.05). ^a,b,c,d,e,f,g^Values within a row of creatinine and BUN between groups were different (*P* < 0.05).

## Data Availability

The data used to support the findings of this study are available from the corresponding author upon request.

## References

[1] Matsumoto T., Kitagawa T., Ohta T. (2019). Structures of triterpenoids from the leaves of *Lansium domesticum*. *Journal of Natural Medicines*.

[2] Lazuardi M., Suharjono S., Chi-Hsien C. (2021). Encapsulation of progesterone-like compounds in 10% liposome increases their concentration in rats administered an injectable dosage form of these compounds. *Kafkas Universitesi Veteriner Fakultesi Dergisi*.

[3] Loef M., Walach H. (2020). Quality of life in cancer patients treated with mistletoe: a systematic review and meta-analysis. *BMC Complementary Medicine and Therapies Complementary Medicine and Therapies*.

[4] Ma L., Phalke S., Stévigny C., Souard F., Vermijlen D. (2020). Mistletoe-extract drugs stimulate anti-cancer V*γ*9V*δ*2 T cells. *Cells*.

[5] Mochamad L., Hermanto B., Hestianah E. P. (2019). Determination of progesterone compounds in the crude methanol extract of benalu duku leaves. *Vet World*.

[6] Hardiyanti R., Marpaung L., Adnyana I. K., Simanjuntak P. (2019). Biochemical evaluation of duku’s mistletoe leave (*Dendrophtho epentandra (L.) Miq)* extract with antidiabetic potential. *Rasayan Journal of Chemistry*.

[7] Lopresti A. L., Smith S. J., Malvi H., Kodgule R. (2019). An investigation into the stress-relieving and pharmacological actions of an ashwagandha (*Withania somnifera*) extract: a randomized, double-blind, placebo-controlled study. *Medicine (Baltimore)*.

[8] Adoho A. C. C., Konm B. B. S., Olounladé P. A. (2022). Phytochemistry and larval toxicity of Ipomea asarifolia, Commenlina diffusa, Acalypha ciliata and Eleusine indica againts Artemia salina. *International Journal of Veterinary Science*.

[9] Watson C. J., Whitledge J. D., Siani A. M., Burns M. M. (2021). Pharmaceutical compounding: a history, regulatory overview, and systematic review of compounding errors. *Journal of Medical Toxicologymedical toxicology*.

[10] Saskianti T., Nugraha A. P., Prahasanti C., Ernawati D. S., Suardita K., Riawan W. (2020). Immunohistochemical analysis of stem cells from human exfoliated deciduous teeth seeded in carbonate apatite scaffold for the alveolar bone defect in Wistar rats (*Rattus novergicus*). *F1000Research*.

[11] Smalheiser N. R., Graetz E. E., Yu Z., Wang J. (2021). Effect size, sample size and power of forced swim test assays in mice: guidelines for investigators to optimize reproducibility. *PLoS One*.

[12] Reilly S. M., Cheng T., DuMond J. (2020). Method validation approaches for analysis of constituents in ENDS. *Tobacco Regulatory Science*.

[13] Munien C., Viriri S., Viriri S. (2021). Classification of hematoxylin and eosin-stained breast cancer histology microscopy images using transfer learning with efficient nets. *Computational Intelligence and Neuroscience*.

[14] Ebbo A. A., Sani D., Suleiman M. M., Ahmad A., Hassan A. (2020). Acute and sub-chronic toxicity evaluation of the crude methanolic extract of *Diospyros mespiliformis* hochst ex a. Dc (*ebenaceae*) and its fractions. *Toxicology Reports*.

[15] Cavaliere C., Capriotti A. L., La Barbera G., Montone C., Piovesana S., Lagana A. (2018). Liquid chromatographic strategies for separation of bioactive compounds in food matrices. *Molecules*.

[16] Qu L., Ruan J., Wu S. (2018). Separation and bioactive assay of 25*R/S*-spirostanol saponin diastereomers from *yucca schidigera* roezl (Mojave) stems. *Molecules*.

[17] Dixit S., Maurya P., Srivastava M. (2019). Quantitation of dietary dihydrochalcones in Indian crabapple *(Malus sikkimensis)* using validated high-performance liquid chromatography. *Journal of Chromatographic Science*.

[18] Sarkis N., Sawan A. (2022). Development and validation of derivative UV spectroscopic method for simultaneous estimation of nicotinamide and tretinoin in their binary mixtures and pharmaceutical preparations. *BMC Chemistry*.

[19] Kamran M., Khan M. R., Khan H. U., Abbas M., Iqbal M., Nazir A. (2018). Phytochemical and cytotoxic evaluation of Medicago monantha: in vivo protective potential in rats. *Biomedicine & Pharmacotherapy*.

[20] Thammitiyagodage M. G., de Silva N. R., Rathnayake C. (2020). Biochemical and histopathological changes in Wistar rats after consumption of boiled and un-boiled water from high and low disease prevalent areas for chronic kidney disease of unknown etiology (CKDu) in north Central Province (NCP) and its comparison with low disease prevalent Colombo, Sri Lanka. *BMC Nephrology*.

[21] Alomar M. Y. (2020). Physiological and histopathological study on the influence of *Ocimum basilicum* leaves extract on thioacetamide-induced nephrotoxicity in male rats. *Saudi Journal of Biological Sciences*.

[22] Ghante M. H., Jamkhande P. G. (2019). Role of pentacyclic triterpenoids in chemoprevention and anticancer treatment: an overview on targets and underling mechanisms. *Journal of Pharmacopuncture*.

[23] Ibrahim K. E., Al-Mutary M. G., Bakhiet A. O., Khan H. (2018). Histopathology of the liver, kidney, and spleen of mice exposed to gold nanoparticles. *Molecules*.

[24] Sun T., Dong W., Jiang G. (2020). *Cordyceps militaris* improves chronic kidney disease by affecting TLR4/NF-*κ*B redox signaling pathway. *Oxidative Medicine and Cellular Longevity*.

[25] Ahmadian S., Sheshpari S., Mahdipour M. (2019). Toxic effects of VCD on kidneys and liver tissues: a histopathological and biochemical study. *BMC Research Notes*.

[26] Loha M., Mulu A., Abay S. M., Ergete W., Geleta B. (2019). Acute and subacute toxicity of methanol extract of *syzygium guineense* leaves on the histology of the liver and kidney and biochemical compositions of blood in rats. *Evidence-based Complementary and Alternative Medicine*.

[27] Haedrich J., Stumpf C., Denison M. S. (2020). Rapid extraction of total lipids and lipophilic POPs from all EU-regulated foods of animal origin: smedes’ method revisited and enhanced. *Environmental Sciences Europe*.

[28] Joshi D. R., Adhikari N. (2019). An overview on common Organic solvents and their toxicity. *Journal of pharmaceutical Research International*.

